# The homeobox protein VentX reverts immune suppression in the tumor microenvironment

**DOI:** 10.1038/s41467-018-04567-0

**Published:** 2018-06-05

**Authors:** Yi Le, Hong Gao, Ronald Bleday, Zhenglun Zhu

**Affiliations:** 1 Department of Medicine, Brigham and Women’s Hospital, Boston, Massachusetts USA; 2 Department of Medicine, Tufts Medical Center, Boston, Massachusetts USA; 3 Department of Surgery, Brigham and Women’s Hospital, Boston, Massachusetts USA

## Abstract

Immune suppression in the tumor microenvironment (TME) is a central obstacle to effective immunotherapy. Tumor-associated macrophages (TAMs) are key components of the TME. Although TAMs have been viewed as an ideal target of intervention to steer immunity in cancer treatment, the approach has been hampered by the lack of knowledge of how TAM plasticity is controlled by cell intrinsic factors. VentX is a homeobox protein implicated in proliferation and differentiation of human hematopoietic and immune cells. Using clinical samples obtained from cancer patients, we find that VentX expression is drastically reduced in TAMs. We show here that VentX promotes M1 differentiation of TAMs, and that VentX-regulated TAMs, in turn, revert immune suppression at the TME. Using a NSG mouse model of human colon cancers, we demonstrate that VentX regulates TAM function in tumorigenesis in vivo. Our findings suggest a mechanism underlying immune suppression at TME and potential applications of VentX-regulated TAMs in cancer immunotherapy.

## Abstract (web summary)

Tumour associated macrophages (TAMs) polarize into either pro-tumor or anti-tumor phenotypes. Here the authors show that the homeobox protein VentX is downregulated in clinical samples of colorectal cancer and regulates TAMs plasticity with its forced re-expression converting TAMs into an anti-tumor phenotype.

## Introduction

The role of immunity in oncogenesis has been long appreciated and increasingly exploited. Nevertheless, the efficacy of cancer immunotherapy remains limited, especially for solid tumors. Immune suppression at the tumor microenvironment (TME) has been accounted for cancer evasion of immune destruction and regarded as a potential venue of intervention^1^. However currently, mechanisms that underlie immune suppression at TME remained largely elusive^2, 3^. Tumor-associated macrophages (TAMs) are key components of TME^4^ and have been implicated in growth, invasion, and metastasis of nearly all tumors. Derived from circulating monocytes, TAMs display a broad spectrum of phenotypes, ranging from the pro-inflammatory M1-like phenotype in early stages of some tumors to the M2-like phenotype in most advanced tumors^4, 5^. The M2-like TAMs show an elevated expression of interleukin (IL)-10, matrix metallopeptidase (MMP), and vascular endothelial growth factor (VEGF), but decreased expression of pro-inflammatory tumoricidal cytokines, cytotoxic inducible nitric oxides (iNOs) and reactive oxygen intermediates (ROIs), and are thought to be supportive of tumor growth^6^. Besides their functions in promoting tumorigenesis, TAMs also help generate a pro-tumor immune suppressive milieu at TME by altering the composition and function of tumor-infiltrating lymphocytes (TILs)^3, 6^. The plasticity of TAMs has been well recognized. It has been proposed that by converting the pro-tumor M2-like TAMs into the anti-tumor M1-like phenotype, the TAMs might be converted into an effective modality of anti-tumor therapy^7, 8^. Nevertheless, this potential application of modulating TAM function in cancer treatment has been hampered by our ignorance of how TAMs plasticity is controlled by cell intrinsic factors^5, 9^.

Dorsoventral axis formation represents coordinated cell proliferation and differentiation during early vertebrate embryogenesis. In trying to understand the molecule basis of the process, our recent work led to the appreciation of the *Xenopus* homeobox protein Xom, a unstable protein of the ventral BMP4 signaling pathway^10^, as a novel  lymphoid enhancing factor/T-cell factor (LEF/TCF)-associated factor that antagonizes β-catenin of the dorsal Wnt signaling pathway during dorsoventral polarization of *Xenopus* embryos^11^. To explore the potential clinical relevance of the findings, we performed sequence homology search and functional analysis, which led to the identification of human homeobox protein VentX as a human homolog of Xom^12, 13^. We found that VentX is primarily expressed in hematopoietic cells and controls proliferation and differentiation of hematopoietic cells from early ontogenesis to terminal differentiation^12, 14–16^. Interestingly, comparative genomic studies showed that VentX is preserved in primates and human but lost in mice since the evolutionary divergence of rodent and primate lineages^17^. Using an in vitro culture of human monocyte-derived macrophage model, we found that VentX promotes and is required for M1 but not M2 activation^14^. These findings inspired us to explore whether VentX has a role in regulating plasticity and function of TAMs.

Using clinical samples obtained from primary colorectal cancer patients, our current studies showed that VentX expression is drastically reduced in TAMs in comparison with its expression in macrophages isolated from normal mucosa of the same patients. We found that TAM VentX expression profile correlates with TAM phenotypes, and that ectopic expression of VentX converted the M2-like phenotype of TAMs into M1-like phenotype. Moreover, we found that VentX-regulated TAMs revert immune suppression at TME by inhibiting regulatory T-cell (Treg) differentiation and promoting CD8 TIL activation. Using a NOD scid γ (NSG) mouse model of patient-derived xenograft (NSG-PDX) of colon cancers, we showed that VentX-modulated-TAMs function in tumorigenesis in vivo. Taken together, our studies suggested a key role of VentX in regulating TAM plasticity and immune status at TME. Targeting VentX, therefore, may open novel venue of cancer immunotherapy.

## Results

### VentX expression is decreased in TAMs

**Fig. 1:**
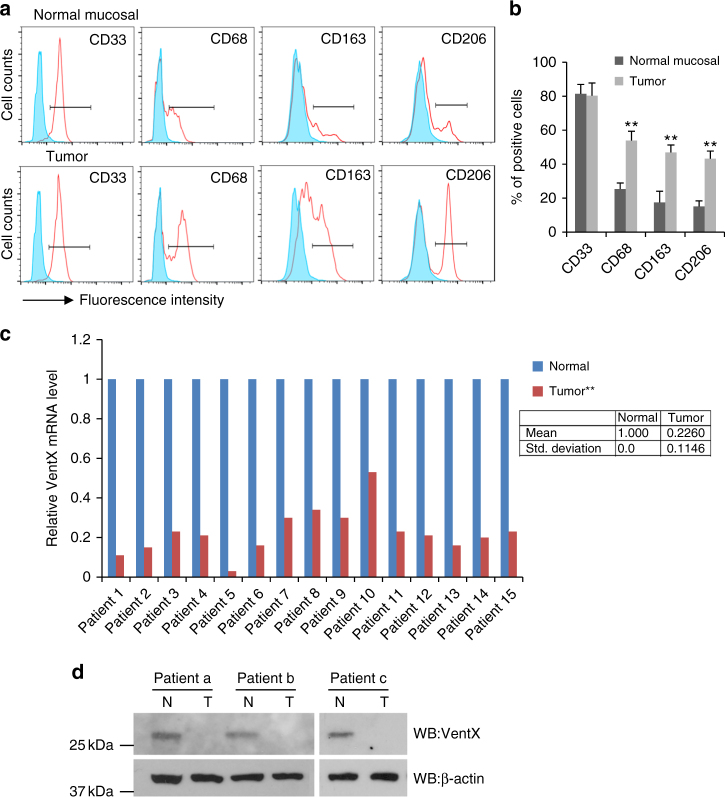
VentX expression is downregulated in TAMs. **a**, **b** FACS analysis and the percentage of surface expression of CD33, CD68, CD163, and CD206 on macrophages isolated from normal mucosa and tumors. Shaded peaks indicated isotope control. Representative figures of five independent experiments were shown, *n* = 5. Error bars represent SD and paired Student’s *t*-test was performed. **p *<* 0.01. **c** Paired comparison of RT-PCR measurement of VentX mRNA expression in macrophages isolated from normal control tissues and TAMs of 15 patients. The relative VentX mRNA expression levels in normal macrophages were arbitrarily designated as 1. Paired Student’s *t*-test was performed, **p *<* 0.01 TAMs vs. normal macrophages. Data shown in the box are mean ± SD of 15 patients. **d** Western blot analysis of endogenous VentX protein levels in macrophages isolated from normal control tissues and TAMs of three patients

In advanced tumors, TAMs display a pro-tumor M2-like phenotype^7, 18, 19^. The plasticity of TAMs has been well appreciated, however, the transcriptional machinery that controls the TAM polarization remains largely unknown. Using TAMs isolated from discarded specimens from colon cancer resection as well as macrophages from normal mucosa 10 cm away from the tumor sites, we sought to explore the potential involvement of VentX in TAM plasticity. Consistent with prior findings, we showed that TAMs express significant higher levels of cell surface markers associated with M2 phenotypes, such as CD163 and CD206^20–23^ (Fig. 1a, b). The elevated expression of M2 surface markers was accompanied by a decrease of M1 surface markers, such as CD40 and7 CD80 (Supplementary Fig. 1); however, there was no significant difference in the expression of non-discriminating myeloid marker CD33 in TAMs and control macrophages (Fig. 1a, b). Similar to the cell surface markers, there is an increased expression of markers associated with M2 differentiation but decreased expression of M1 markers in TAMs (Supplementary Fig. 2). To determine whether VentX has a role in TAM plasticity, we quantified VentX expression in TAMs by quantitative reverse-transcription PCR (qRT-PCR) and found that, in comparison with its expression in control macrophages, VentX expression is decreased about 77% in TAMs (Fig. 1c). The decreased expression of VentX in TAMs was further verified by western blot analysis, using VentX-specific antibodies (Fig. 1d).

### VentX drives TAM towards M1 phenotype

**Fig. 2:**
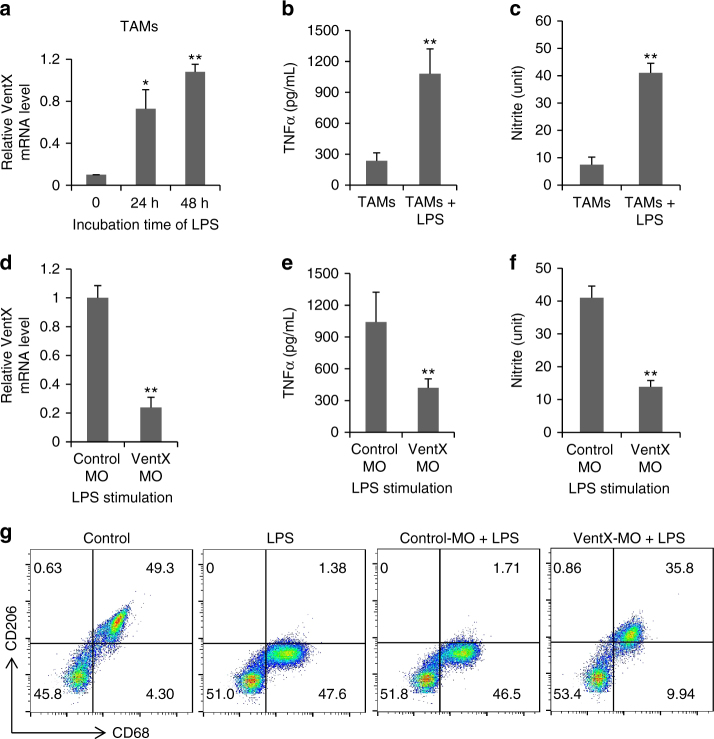
VentX regulates TAM plasticity. **a** VentX expression in TAMs after LPS stimulation. Isolated TAMs were cultured in RPMI medium and stimulated with LPS. VentX mRNA levels were measured with real-time PCR at indicated times. **b** TNF-α and **c** nitrate level from TAMs after LPS treatment were determined by ELISA and Griess reagent. **d** Effects of VentX-MO on VentX expression. Isolated TAMs were transfected with MO oligonucleotides targeting VentX or control. Twenty-four hours post transfection, LPS was added to culture medium. The cells were collected 48 h later and VentX expressions were determined by real-time RT-PCR. **e** TNF level and **f** nitrate level from VentX-MO- or control-MO-treated TAMs after LPS stimulation. Data shown are mean ± SD of three independent experiments and paired Student’s *t*-test was performed. *p *<* 0.05, **p *<* 0.01. **g** Effects of VentX-MO or control-MO on percentage of CD206+CD68+ TAMs after LPS stimulation. Representative data of at least three independent experiments were shown

To determine whether VentX is involved in TAM plasticity, we examined the correlation of VentX expression with TAM phenotypes. Using lipopolysaccharide (LPS) as an M1 phenotype inducer^24^. we found that VentX expression is significantly elevated in TAMs after them being exposed to LPS (Fig. 2a). The elevated expression of VentX is accompanied by elevated expression of M1 markers in TAMs, including the secretion of inflammatory cytokines and cytotoxic iNOs (Fig. 2b, c). Similar to LPS, we found that TAMs can also be activated by pro-inflammatory cytokines, such as interferon-γ (IFNγ) (Supplementary Fig. 3). To determine whether VentX has a required role in TAM plasticity, we examined the effects of knockdown VentX on TAM phenotypes. As shown in Fig. 2d, treatment of TAM with VentX morpholino oligos (VentX-MO) led to around 80% reduction of VentX expression. The decreased VentX expression, which is verified by western blot analysis (Supplementary Fig. 4), is accompanied by decreased secretion of inflammatory cytokines and cytotoxic iNOs (Fig. 2d–f). Corresponding to the changes of M1 phenotype, there is also a change of the M2 phenotype. As shown in Fig. 2g, we found that the effect of LPS on the expression of CD206, a M2 marker highly expressed in TAMs (Supplementary Fig. 1a)^25^, was abolished by VentX-MO treatment.

**Fig. 3:**
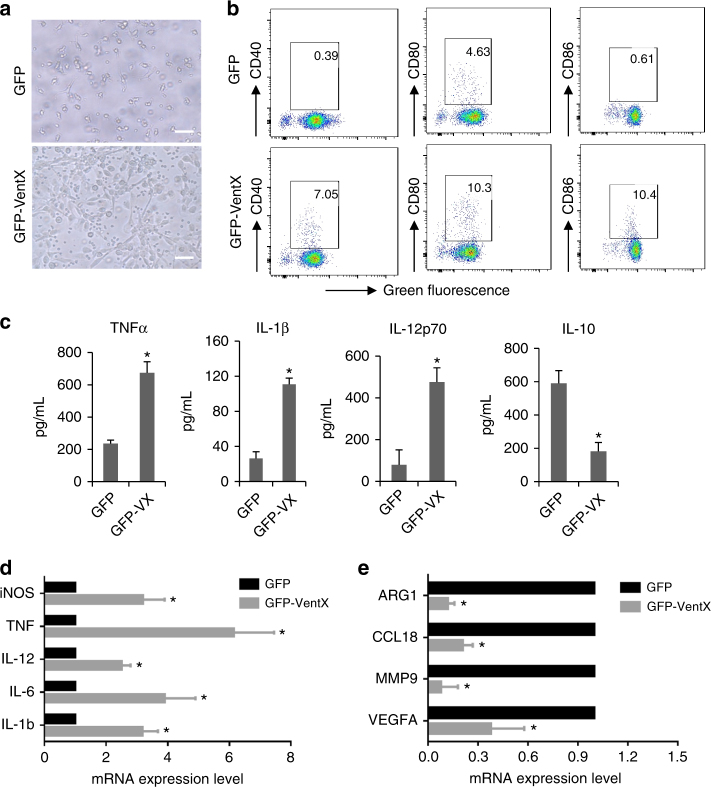
VentX polarizes TAMs towards M1 phenotype. **a** Morphology of TAMs transfected with GFP or GFP-VentX. Scale bar: 20 μm. **b** Surface expression of CD40, CD80, and CD86 in TAMs transfected with GFP or GFP-VentX as determined by flow cytometry analysis. Representative data of three independent experiments were shown. **c** ELISA measurement of secretion of pro-inflammatory cytokines in TAMs transfected with GFP and GFP-VentX. Results represent mean ± SD of four independent experiments and paired Student’s *t*-test was used. Expression of M1 (**d**) and M2 (**e**) macrophage markers in TAMs transfected with GFP or GFP-VentX as determined by qRT-PCR. Data shown are mean ± SD of three independent experiments and paired Student’s *t*-test was performed. *p *<* 0.05

The correlation between VentX expression levels and TAM phenotypes prompted us to explore the potential direct effect of VentX on TAM phenotype. Using transfection studies, we found that TAMs transfected with GFP-VentX displayed a characteristic M1 morphology with elongated/fibroblast-like cell shape, whereas there was no such changes in TAMs transfected with the control green fluorescent protein (GFP) (Fig. 3a). In comparison with the control GFP-transfected TAMs, the surface expression of M1 marker CD40, CD80, and CD86 was significantly increased in TAMs transfected with GFP-VentX (Fig. 3b). In addition, the secretion of pro-inflammatory cytokines tumor necrosisfactor-α (TNFα), IL-1β, and IL-12 were significantly increased, whereas secretion of the M2 cytokine, IL-10, was significantly reduced (Fig. 3c). Using qRT-PCR analysis, we showed a characteristic increased expression of of M1 genes, such as IL-1β, IL-6, IL-12, TNF-α, and iNOs (Fig. 3d), and decreased expression of M2 genes, such as CCL18, MMP9, VEGFA, and Arg1 in TAMs transfected with GFP-VentX (Fig. 3e). In comparison, there was no significant changes of non-discriminating macrophages markers, such as the CD33 and CD68 upon the transfection of GFP-VentX in TAMs (Supplementary Fig. 5a, 5b). The changes of cell surface markers are associated with changes of intracellular signaling molecules such as the STAT1 and STAT3, which are associated with M1 and M2 phenotype, respectively (Supplementary Fig. 5c)^26^. Taken together, our data suggested that VentX functions as a master switch of TAM plasticity and drives TAMs toward M1 phenotypes through alternating intracellular signaling pathways involved in the process.

### VentX-regulated TAMs modulate TILs differentiation

**Fig. 4:**
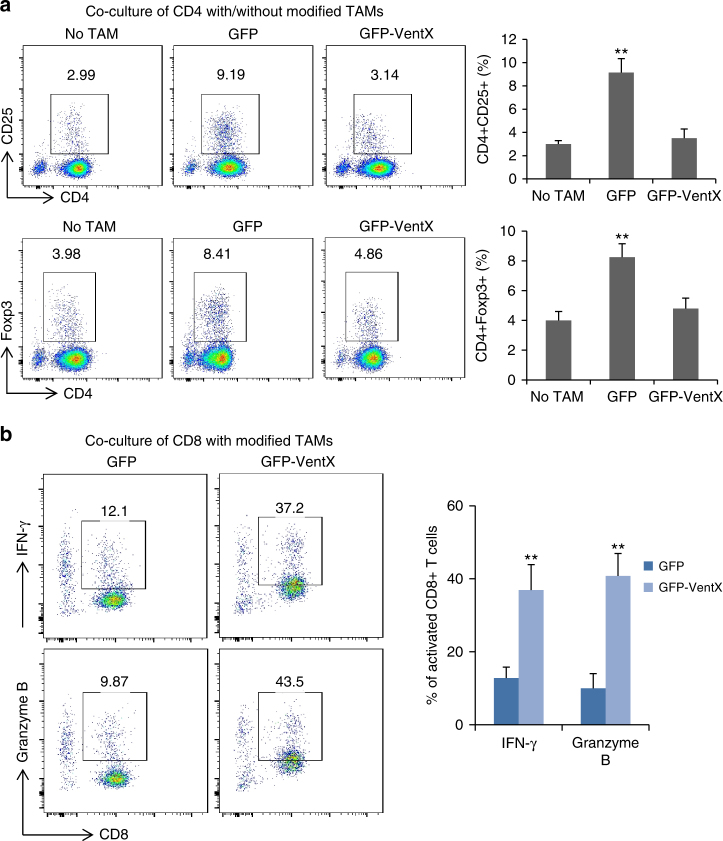
VentX modulates TAM induction of TILs differentiation and activation. **a** VentX modulates TAM induction of T-cell differentiation. CD4+ T cells were incubated with autologus TAMs transfected with GFP or GFP-VentX for 5 days. Differentiation of the CD4+ T cells were determined by CD25 and Foxp3 analysis. The results were mean ± SD of five independent experiments. **b** VentX modulates TAM effects on CD8 activation. CD8 TILs isolated from tumors were incubated with autologus TAMs transfected with GFP or GFP-VentX for 5 days. CD8+TIL activation was measured by FACS analysis of IFNγ and granzyme B. Data shown are mean ± SD of three independent experiments and paired Student’s *t*-test was performed. *p *<* 0.05, **p *<* 0.01

TILs have key roles in anti-tumor immunity^3^. The regulatory CD4+CD25+ Treg and tumoricidal CD8+ TILs are key components of TME. The ratio of CD4+CD25+ Tregs and CD8+ TILs, as well as functional defects of CD8 TILs, have been implicated in pathogenesis and prognosis of solid tumors^6, 27–30^. Consistent with prior findings^31^, we found that there was a significantly increased number of the CD4+CD25+/CD4+Foxp3+ Treg cells and a significantly decreased number of CD8+ TILs in tumor tissues (Supplementary Fig. 6 and 7). As CD4+CD25+/CD4+Foxp3+ Treg cells are derived from CD4+ T cells, to explore the potential involvement of VentX-regulated TAMs in compositions of TILs, we sought to determine whether VentX-regulated TAMs modulate CD4+ T-cell differentiation. To attend our goal, CD4+ T cells isolated from peripheral blood were co-cultured with autologus TAMs transfected with either GFP-VentX or control GFP for 5 days. As shown in Fig. 4a, co-culture of CD4+ T cells with TAMs led to significant induction of CD4+CD25+/CD4+Foxp3+ cells. In contrast, ectopic expression of VentX in TAMs abolished the induction. To corroborate the functional relevance of the findings, CD4+ T cells isolated from normal tissues were co-cultured with autologus TAMs transfected with either GFP-VentX or control GFP for 5 days. We found that TAMs induced T-cell expression of inhibitory IL-13, but the induction function was abolished by ectopic expression of VentX (Supplementary Fig. 8). To further determine whether VentX-regulated TAMs modulate TIL function, CD8+ TILs were co-cultured with TAMs transfected with GPF-VentX or control GFP. As shown in Fig. 4b, co-culture of CD8+ TILs with VentX-modified TAMs led to significant activation of CD8+ TILs, as indicated by CD8+ activation markers, such as IFNγ and granzyme B. Taken together, our data suggested that VentX-regulated TAMs modulate TIL differentiation and function.

### VentX-regulated TAMs modulate composition of TILs in TME

**Fig. 5:**
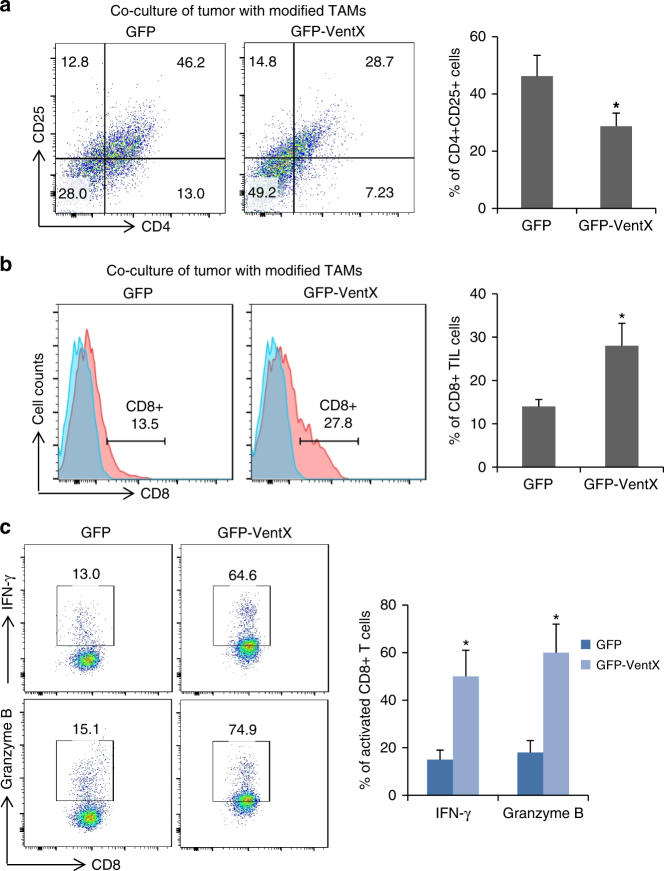
VentX-regulated TAMs modulate composition and function of TILs in tumor microenvironment. **a**, **b** Composition of CD4+CD25+ Tregs (**a**) and CD8+ TILs (**b**) in tumors after en bloc co-culture of tumor tissues with autologus TAMs transfected with GFP or GFP-VentX for 5 days. **c** CD8 TILs were isolated from tumor tissues after being incubated with autologus TAMs transfected with GFP or GFP-VentX for 5 days. CD8+TIL activation was measured by FACS analysis of IFNγ and granzyme B. Data shown are mean ± SD of three independent experiments and paired Student’s *t*-test was performed. *p *<* 0.05

Our findings that VentX-regulated TAMs modulate differentiation and function of T cells prompted us to test the hypothesis that VentX-regulated TAMs control immune status at TME by modulating the differentiation and function of TILs. To test this hypothesis, en bloc primary tumor tissues were co-cultured with autologous TAMs transfected with GPF-VentX or control GFP for 5 days. CD4+CD25+Treg cells and CD8+ TILs were then isolated and quantified by flow cytometry, following established protocols (Supplementary Fig. 9)^32, 33^. As shown in Fig. 5a, b, co-culture of the tumors with GFP-VentX-modified TAMs led to a significant decrease of the CD4+CD25+Tregs and a significant increase of the CD8+ TILs. In addition to the significant increase in the number of CD8+ TILs, there is a significant increase in the expression of IFNγ and granzyme B in CD8+ cells in tumor tissues after being incubated with VentX-modified TAMs (Fig. 5c).

### VentX regulates TAM function and tumorigenesis in vivo

**Fig. 6:**
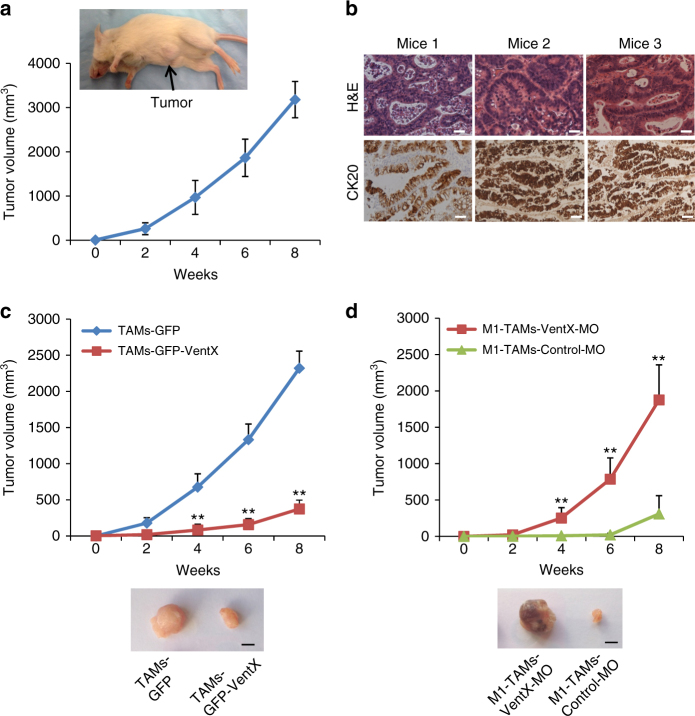
VentX-regulated TAMs modulate tumorigenesis in vivo. **a** A NSG-PDX model of human colon cancers. Small pieces of colon cancer tissues were transplanted into subcutaneous space on the abdominal side of NSG mice and the growth of the tumors were observed for 8 weeks. Data shown were results of seven independent replicates of the experiments. **b** H&E and CK20 staining of tumors resected from three NSG-PDX mice. Scale bar: 50 μm. **c** Growth curve of tumors in NSG-PDX mice treated with TAMs transfected with GFP-VentX or control GFP. Results shown represent mean ± SD of three independently replicated experiments, *n* = 3, and paired Student’s *t*-test was performed, **P < 0.01. **d** Growth curve of tumors in NSG-PDX mice treated with M1-TAMs transfected with VentX-MO or control-MO. Results shown represent mean ± SD of seven independently replicated experiments, *n* = 7, and paired Student’s *t*-test was performed, **P < 0.01. Photos of excised tumors at the end of experiments were shown. Scale bar: 5 mm

Converting the M2 phenotype of TAM into the M1 phenotype has been viewed as a promising venue for cancer treatment^5, 8^. Our findings that VentX promotes M1 polarization of TAM prompted us to explore whether VentX-modulated TAM plasticity has a role in tumorigenesis in vivo. As VentX does not have a mouse homolog^17^, to achieve our goal, we adapted a NSG mouse model, which support heightened engraftment of human hematopoietic cells^34^. Following established protocol^35^, a NSG-PDX of human colon cancers was generated by engrafting small pieces of primary human colon cancer tissues into subcutaneous space on the abdominal side of the NSG mice. The growth of the tumors in the NSG mice was observed for 8 weeks (Fig. 6a). The tumors were then dissected out and sectioned and the growth of human colon cancers was confirmed by hematoxylin and eosin (H&E) and CK20 staining (Fig. 6b). To test the potential effects of VentX-regulated TAMs on tumor growth 1 week post implantation of fragments of colon cancers, the NSG-PDX mice were tail-vein injected with TAMs transfected with GFP-VentX or control GFP. As shown in Fig. 6c, we found that, in comparison with the GFP-transfected TAMs, GFP-VentX-transfected TAMs exerted strong inhibition on tumor growth in the NSG-PDX mice. To further determine whether the inhibition of tumorigenesis in the NSG-PDX mice is related to VentX-regulated TAM polarity 1 week post implantation of fragments of colon cancers, the NSG-PDX mice were tail-vein injected with in vitro M1-differentiated TAMs transfected with either VentX-MO or control-MO. Consistent with the results of over expression studies, the M1-TAMs transfected with control-MO exerts strong inhibition of tumor growth, but the inhibition was abolished by knocking-down VentX expression with VentX-MO (Fig. 6d).

## Discussion

Tumor immunology is now a promising field for exploration of pathogenesis and treatment of cancers. Macrophages have executor role in immunity and have been recognized as key components of tumor mass. The search for factors that can be manipulated to steer macrophage plasticity has proved to be a fundamental challenge. Signaling pathways such as the JAK/STAT1, JNK/STAT6, AKT1/AKT2, and PI3Kγ, transcriptional regulators such as PPARγ and PPARδ, NFκB, C/EBP, and IRFs, as well as microRNAs such as microRNA-155 and miR-142-3p have been shown to modulate macrophage differentiation^9, 24, 36, 37^. Nevertheless, whether these factors can be targeted to turn TAMs into a tumoricidal cells remains unclear. As a unique human hematopoietic transcriptional factor that does not have a murine homolog, VentX was found to have essential role in controlling proliferation and differentiation of hematopoietic cells and function as a p53-independent tumor suppressor^12, 15, 16, 38–40^. Data of our current study suggested VentX controls TAM plasticity, which in turn, reverts immune suppression at the TME through regulating TIL differentiation and function, which was supported by both in vitro and in vivo studies (Figs. 4, 5 and Supplementary Fig. 11). Interestingly, consistent with prior findings that macrophages may affect T-cell differentiation indirectly^41^, we found that TAMs do not need to accumulate in significant numbers inside tumor tissues to exert its function (Supplementary Fig. 12). Our findings revealed a novel mechanism underlying immune suppression at TME and suggested the potential application of VentX-regulated TAMs as a novel modality of immunotherapy, especially for solid tumors, which are refractory to current available immunotherapy. Currently, the cause of decreased VentX expression in TAMs and the potential clinical application of VentX-modulated TAMs in cancer treatment remain to be further defined.

## Methods

### Collection of colon tissue samples

A total of 42 patients with colon cancer, who were scheduled for surgical resection at Brigham and Women’s Hospital, were consented to have a portion of tissues and blood collected for research purposes. All patients signed an informed consent document that was approved by the Institutional Review Board of Brigham and Women’s Hospital. The characteristics of colon cancer specimens used for this study were listed in Table 1. Around 5–10 g tissues were collected from tumor mass, or normal mucosa 10 cm away from tumor mass.

**Table 1:** Characteristics of colon cancer patients used for study

Characteristics	Number (*n* = 42)	(%)
**Age**, **years**		
≤ 40	5	11.9
40–60	16	38.1
≥ 60	21	50.0
**Sex**		
Male	24	57.1
Female	18	42.9
**Pathology type**		
Adenocarcinoma	42	100
**Stage**		
I	8	19
II	14	33.3
III	15	35.7
IV	5	11.9

### Preparation of intraepithelial lymphocytes

Lymphocytes were isolated following previously described techniques with modification^14, 42^. In brief, dissected fresh mucosa and tumor mass were rinsed in 10-cm Petri dish with Ca^2+^-free and Mg^2+^-free Hank’s balanced salt solution (HBSS) (Life Technologies) containing 2% fetal bovine serum (FBS) and 2 mM Dithiothreitol (DTT) (Sigma-Aldrich). The mucosa and tumor were then cut into around 0.1 cm pieces by a razor blade and incubated in 5 mL HBSS containing 5 mM EDTA (Sigma-Aldrich) at 37 °C for 1 h, then passed through a gray-mesh (100 μm). The flowthrough contains intraepithelial lymphocytes and epithelial cells and was analysis by a flow cytometer.

### Isolation of macrophages from tumor mass and normal mucosa

Lamina propria mononuclear cells (LPMCs) were isolated following established protocol, which does not lead to activation of macrophages^14, 42, 43^. Briefly, normal mucosa and tumor tissues were rinsed with HBSS and then incubated in HBSS (with Ca^2+^ and Mg^2+^), containing 2% FBS, 1.5 mg/mL Collagenase D (Roche), 0.1 mg/mL Dnase I at 37 °C for 1 h. Digested tissues were then passed through a gray-mesh (70 μm) filter. The flowthrough were collected, washed, and resuspended in a RPMI 1640 medium. The cells were layered on Ficoll-Paque Plus media (GE Healthcare), and then centrifuged at 2000 r.p.m. for 30 min without brake. LPMCs at the interface were collected. Normal mucosal macrophages and TAMs were further purified from LPMCs using EasySep™ Human Monocyte/Macrophage Enrichment kit without CD16 depletion (StemCell Technologies), according to the manufacturer’s instructions. The isolation process does not lead to activation of macrophages and the purify of isolated intestinal macrophages was routinely more than 95%^14, 42, 43^. More than 98% of cells isolated by the techniques were viable by propidium iodide staining.

### Transfection assays

Transfection of GFP-VentX and GFP into blood macrophages or TAMs were carried out through lipofectamine 2000 (Life Technologies) according to the manufacturer’s protocol. Forty-eight hours after transfection, cells were filtered through a 70 μm filter for cell sorting. GFP-positive cells were sorted by BD FACSAria II under the Baker Bio-Protect Hood in a sterile condition. After sorting, cells were cultured in RPMI 1640 complete medium.

### VentX knockdown

Colon TAMs or human primary monocytes were transfected with Morpholino oligonucleotides (MO) (Gene Tools, LLC, Philomath, OR) using the Human Monocyte Nucleofector Kit (Lonza, Walkersville, MD) as previously described^14^. Briefly, 5 × 10^6^ cells were resuspended into 100 µl nucleofector solution with 2.5 nmol of either VentX-MO oligonucleotides (VentX-MO: 5′-TACTCAACCCTGACATAGAGGGTAA-3′ or a standard control-MO oligonucleotides and electroporated with the Nucleofector II Device (Lonza). Cells were then immediately removed from the device and incubated overnight with 1 ml pre-warmed Human Monocyte Nucleofector Medium containing 2 mM glutamine and 10% FBS. Cells were then resuspended into complete RPMI medium and treated with appropriate cytokines to induce differentiation into macrophages.

### FACS analysis

Phenotypic analysis of TAMs and other lymphocytes was performed using flow cytometry after immunolabeling of cells with fluorescence dye-conjugated antibodies. The following antibodies were used: Phycoerythrin (PE)-conjugated anti-CD3 (OKT3), -CD25 (BC96), -CD14 (61D3), -CD68 (Y182A), -CD163 (GH161), and -CD206, and fluorescein isothiocyanate (FITC)-conjugated anti-CD4 (RPA-T4) and -CD33 (HIM3-4), and Allophycocyanin (APC)-conjugated anti-CD8 (OKT8) and -CD4 (OKT4) (eBioscience, Inc). Intracellular staining of Foxp3 (236 A/E7), IFNγ (4 S.B3), and Granzyme B (GB11) was performed with PE-conjugated antibodies following the protocol provided by manufacturer. Isotope control labeling was performed in parallel. Antibodies were diluted as recommended by the supplier. Labeled cells were collected on FACScan flow cytometer with Cell-Quest software (BD Biosciences) and analyzed by FlowJo software. Results are expressed as the percentage of positive cells.

### Cytokine measurement

Levels of IL-1β, IL-10, IL-13, TNF-α, and IL-12p70 were quantified using enzyme-linked immunosorbent assay kits obtained from eBiosciences. Analyses were conducted according to the manufacturer’s instructions.

### Quantitative RT-PCR

Total RNA was isolated by the TRIzol reagent (Life Technologies) and RNA amounts were measured by NanoDrop 2000 (Thermo Scientific). An equal amount of RNA was used for first-strand complementary DNA synthesis with SuperScript III First-Strand Synthesis System (Life Technologies) according to the manufacturer’s protocol. To amplify VentX cDNA with conventional PCR, we used the AccuPrime Taq DNA polymerase system (Life Technologies) following the manufacturer’s instructions. Quantitative measurement of VentX and other genes cDNA were carried out with SYBR Green on a LightCycler (480 Real-Time PCR System; Roche). The primers used list in Supplementary Table 1. Relative expression profiles of mRNAs were then calculated using the comparative Ct method (DDCT method).

### Western blot analysis

Western blot analysis was performed as described previously^14^. Briefly, total cells were lysed in 1 × RIPA buffer (Abcam, Inc.) mixed with protease inhibitor cocktails (Cell Signaling Technology). Proteins were resolved by 4–15% Tris-Glycine Gel (Bio-Rad) electrophoresis. Primary antibodies used included GFP (eBioscience 14–6674, 1:1000), VentX (Abcam, Inc. ab105352, 1:500), and β-actin (Cell Signaling Technology 4967, 1:2000).

### Arginase activity and NO assays

Arginase activity was quantified in cell lysates by measuring the production of urea using the QuantiChrom Arginase Assay Kit, following the manufacturer’s instuctions (DARG-200, BioAssays Systems). Nitrite concentrations in culture supernatants were determined using Griess reagent kit (Molecular Probes, Eugene, OR), as described previously^14^.

### Treg cell induction and CD8+ TIL cell activation

Treg cell inductions were performed using previously described methods with modification^44, 45^. Briefly, the blood CD4 cells were enriched by using Easysep human CD4-negative selection kit following the manufacturer’s instructions (StemCell Technologies). GFP-VentX or GFP-transfected TAMs (0.5 × 10^6^) were incubated with 5 × 10^6^ of CD4 in completed RPMI 1640 at 37 °C, 5% CO_2_ for 5 days. Cells were stained with CD4-FITC and CD25-PE, or permeabilized and stained with CD4-FITC and Foxp3-PE, then analyzed by a flow cytometer. For CD8+ TIL activation assay, CD8+ TILs were enriched from intraepithelial lymphocytes by CD8+ T-cell Enrichment Kit (StemCell Technologies). Cells (5 × 10^6^) were then incubated with 0.5 × 10^6^ of GFP-VentX or GFP-transfected TAMs for 5 days, followed by staining and analysis with flow cyotmetry.

### Co-cultures of tumors and TAMs

Tumor mass were washed with 1 × phosphate-buffered saline (PBS) buffer plus antibiotics and then cut into 0.5 cm pieces. Around 80 mg of tissues were mix cultured with 0.5 × 10^6^ of GFP-VentX or GFP-transfected TAMs of same patient in 2 mL of RPMI 1640 completed medium, supplemented with 2.5% antibiotic–antimycotic solution (Cellgro, Manassas, VA). The cultures were incubated at 37 °C, 5% CO_2_ for 5 days. The tissues were then subjected to cell isolation and analyzed by a flow cytometry or immunohistochemistry studies.

### NSG-PDX model of human colon cancers

Animal models of primary human colon cancers were developed as described previously^46^. Briefly, 8-week-old NOD.Cg-*Prkdc*^*scid*^ *Il2rg*^*tm1Wjl*^/SzJ mice (commonly known as NSG mice) were purchased from The Jackson Laboratory and maintained under specific pathogen-free conditions. All animal experiments were approved by the Institutional Animal Care and Use Committee at Harvard Medical School. Colon tumors were cut into around 0.5 cm and surgically seeded in subcutaneous space of abdominal side of NSG mice. TAMs were transfected with VentX-MO or control-MO, and then cultured in M1-Macrophage Generation Media (PromoCell). After 1 week of xenograft, 0.8 × 10^6^ of M1-differentiated TAMs transfected with VentX-MO or control-MO were injected into mice through tail vein. Tumor growth was monitored twice a week and measured by a caliper for 8 weeks.

In vivo tracing of injected TAMs were carried out using carboxyfluorescein succinimidyl ester (CFSE)-stained TAMs, according to the manufacture’s instruction (Molecular Probes). Briefly, half million of TAMs were incubated in 5 mL of 5 μM CFSE staining solution in a 37 °C water bath for 20 min. Cells were then incubated with 20 ml of RPMI 1640 completed medium for 5 min to remove unbounded dye. After centrifugation, cells were dissolved in PBS and then tail-vein injected into NSG mice. Tumor tissues and TAMs were isolated 7 days after injection and TAMs were isolated as CFSE-positive cells.

### Immunohistochemistry

Immunohistochemistry were performed following established protocol^47^. Briefly, tumor or normal mucosa were fixated in formalin (Fisher Scientific Company, Kalamazoo, MI). The tissues were then embedded in paraffin and sectioned. CK20 (Dako, Carpinteria, CA, clone Ks20.8, 1:50) and H&E staining were performed at research pathology cores at Dana-Farber/Harvard cancer center. Microscopic analysis was performed with a Nikon Eclipse Ti fluorescence microscopy. Images were captured at an original magnification of × 40 using a color camera applying the NIS Elements imaging software (Nikon). Brightness and contrast for representative images were adjusted equally among groups.

### Statistical analysis

Student’s *t*-test was used for statistical analysis. Data are presented as mean ± SD. The level of significance is indicated by the *p*-value. In all figures, levels of statistical significance are indicated as *p < 0.05 and **p < 0.01.

### Data availability

The authors declare that all the other data supporting the findings of this study are available within the article and its Supplementary Information files and from the corresponding author upon reasonable request.

## Electronic supplementary material

Supplementary Information
