# Supplementary material for: The homeobox protein VentX reverts immune suppression in the tumor microenvironment
Source: Nat Commun. 2018 Jun 5;9:2175. doi: 10.1038/s41467-018-04567-0 (PMC5988690; doi:10.1038/s41467-018-04567-0)
Supplement: Supplementary file 1 — Supplementary Information [file 41467_2018_4567_MOESM1_ESM.pdf]

The homeobox protein VentX reverts immune suppression in the tumor microenvironment

Le Y *et. al.*

Supplementary Figures and Tables

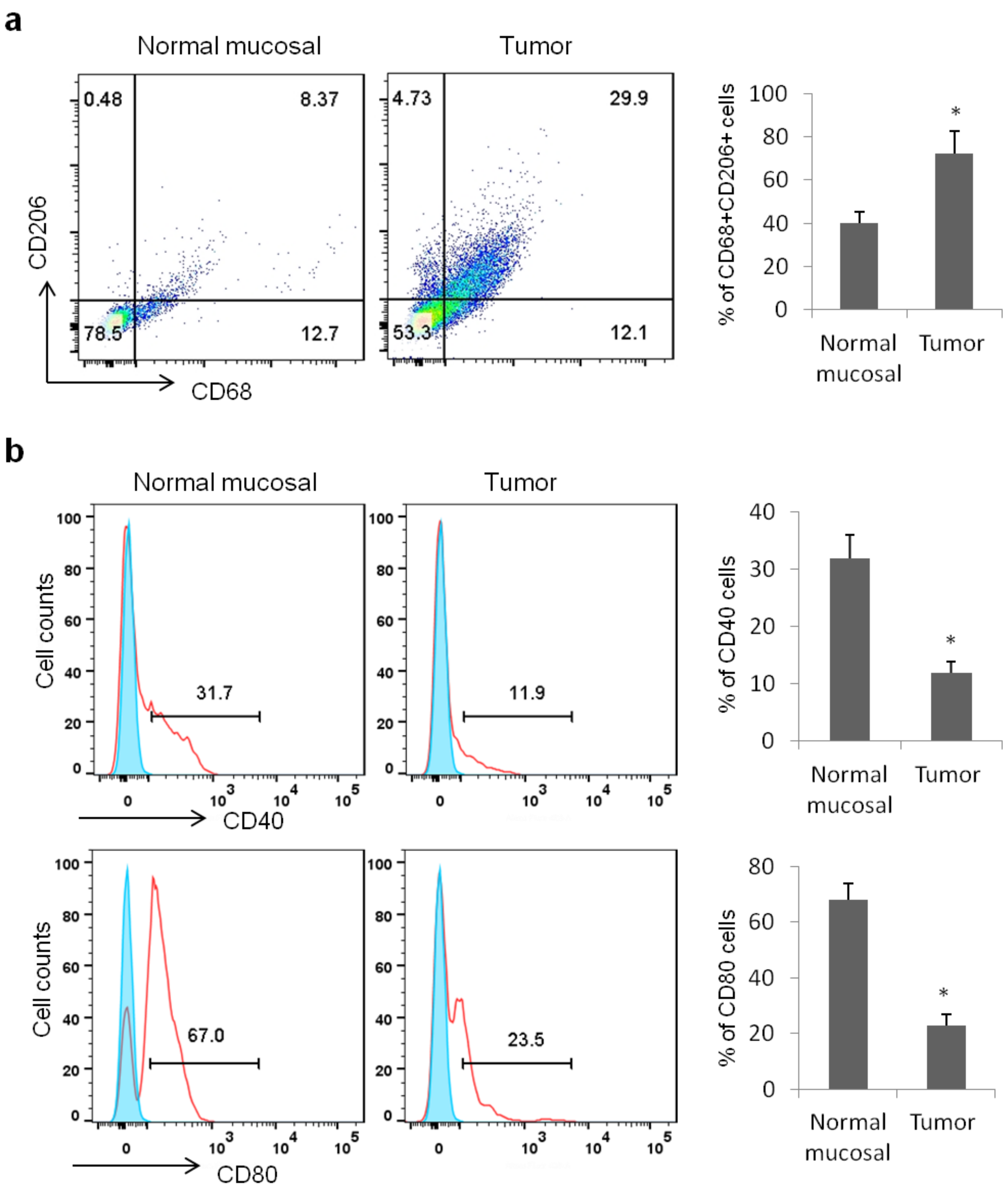

Supplementary Figure 1. TAMs skew towards M2 phenotypes.

Flow cytometry analysis of cell Surface expression of M2 marker CD206 (**a**) and M1 marker CD40 and CD80 (**b**) on the CD68 TAMs. Data shown are the mean ± SD of 3 independent experiments, and paired Student's *t*-test was performed. * p < 0.05.

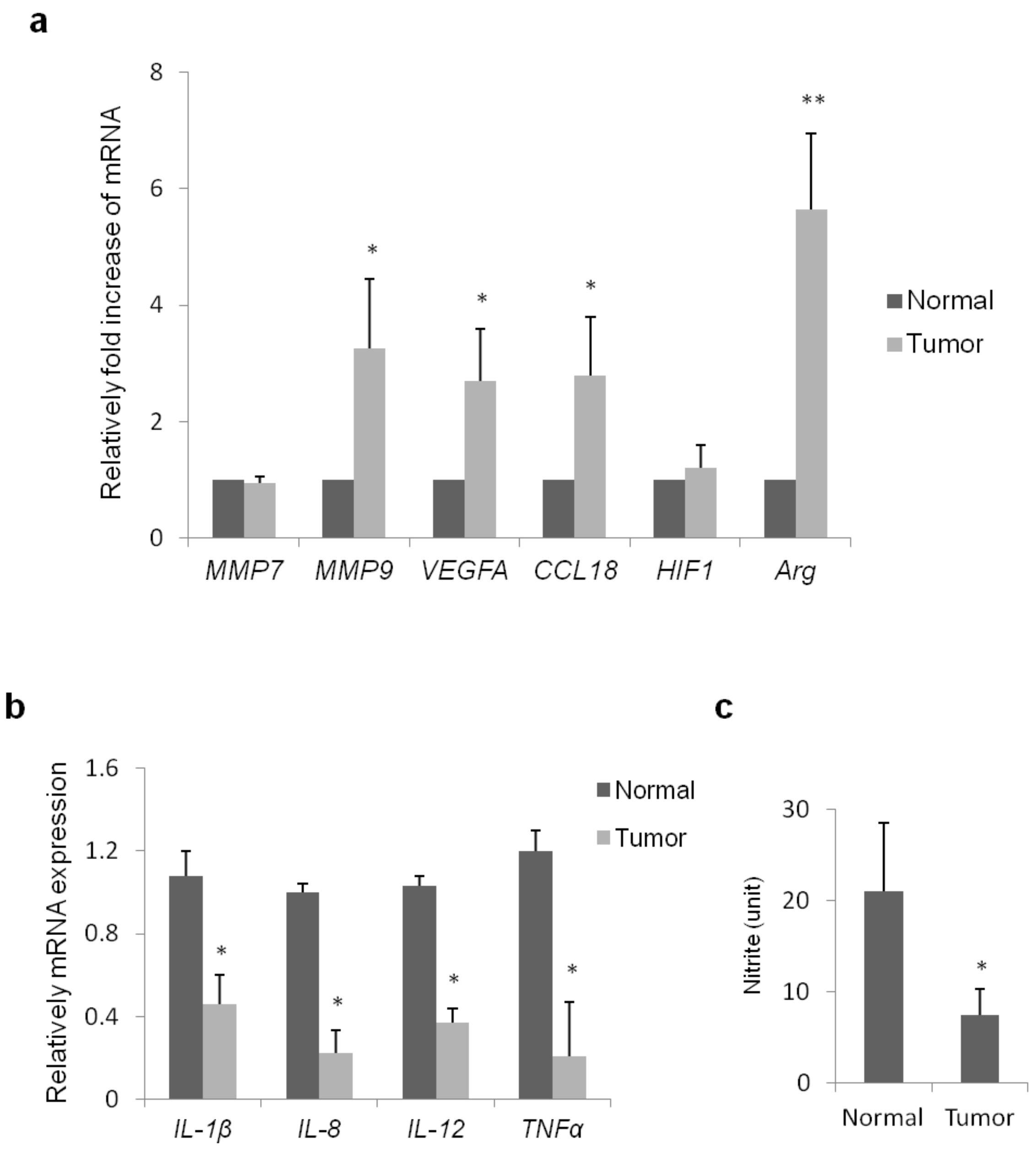

Supplementary Figure 2. Differential expression of M1 and M2 genes in TAMs vs macrophages isolated from normal mucosa.

(**a**) Expression levels of indicated M2 genes in TAMs vs control macrophages as determined by qRT-PCR. (**b**) Expression levels of indicated M1 genes in TAMs vs control macrophages as determined by qRT-PCR**.** (**c**) Nitrate levels of the TAMs and control macrophages. Data shown are mean ± SD of 3 different experiments and paired Student's *t* test was performed. ** indicates $p < 0.01$, *$p < 0.05$.

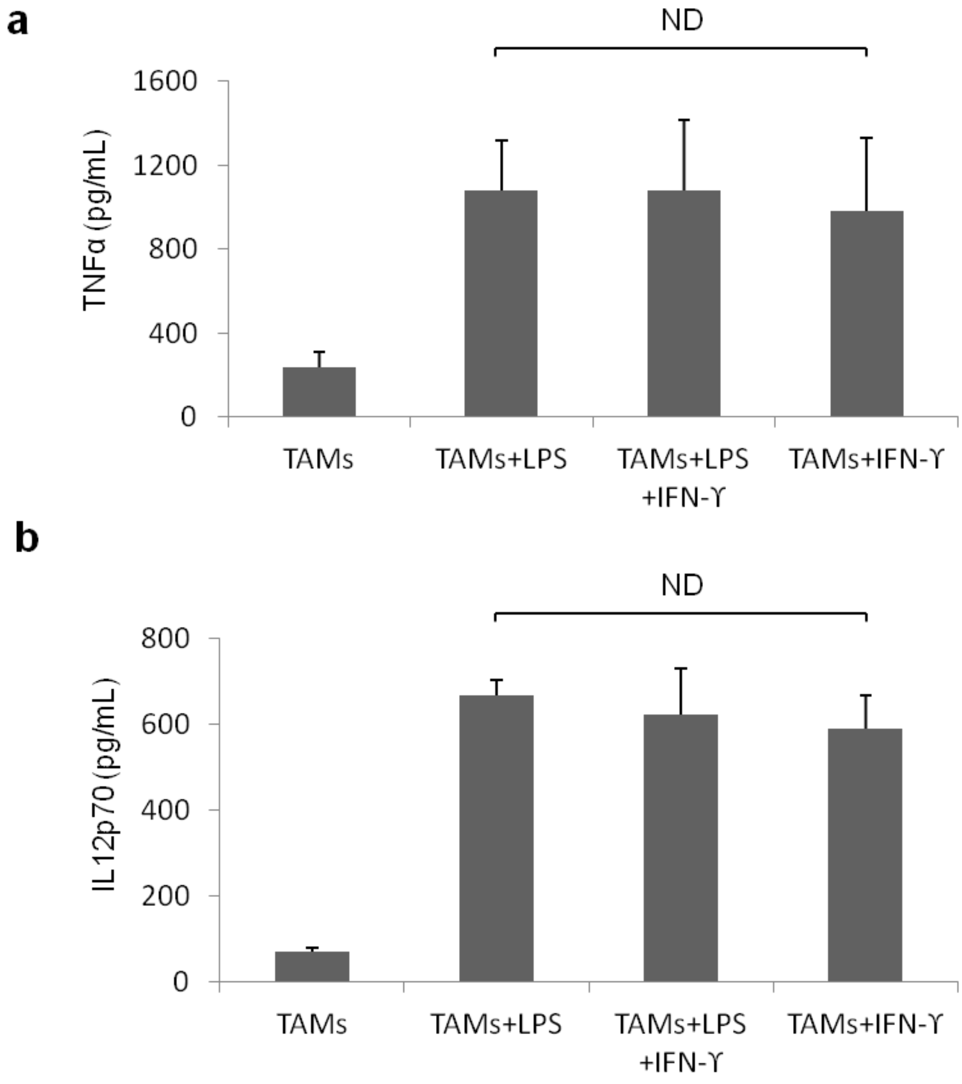

Supplementary Figure 3. Activation of TAMs by pro-inflammatory cytokines.

Isolated TAMs were cultured in RPMI medium and stimulated with LPS, or LPS+IFNγ, or IFNγ. Expression levels of M1 activation markers, TNF-α (**a**) and IL-12p70 (**b**) from the TAMs were determined by ELISA. Data shown are mean ± S.D. of three independent experiments, ND means no significant difference by Student's *t*-test. $p > 0.05$.

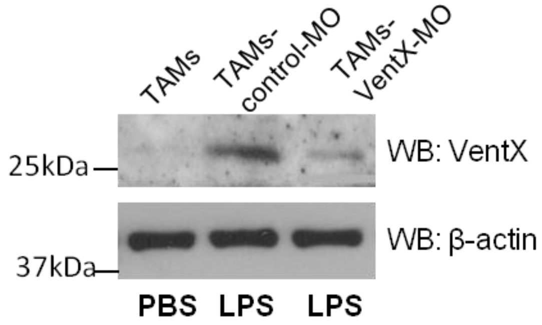

Supplementary Figure 4. Knockdown of VentX expression by VentX-MO.

VentX-MO or control MO transfected TAMs were stimulated with LPS. The effects of the transfection on VentX protein levels were determined by Western blot analysis, using VentX specific antibody. β-actin was used as a loading control.

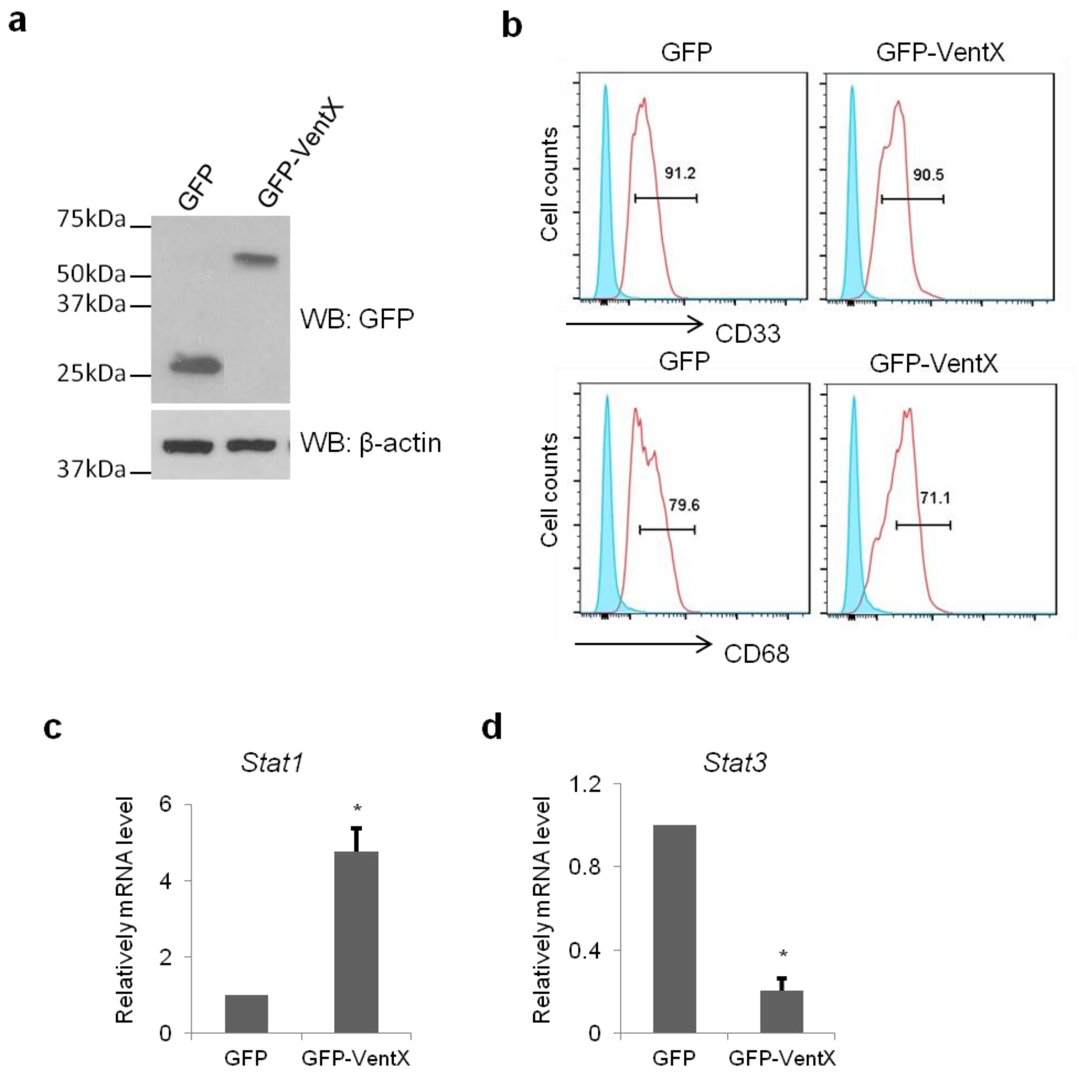

Supplementary Figure 5. Effects of VentX on the expression of intestinal macrophage markers and cell fate determination factors in TAMs.

TAMs were isolated and transfected with plasmids encoding GFP or GFP-VentX as described in material and methods. (**a**) Western blot analysis of GFP and GFP-VentX proteins in TAMs. (**b**) The effects of VentX on the expression of intestinal macrophage markers CD33 and CD68 were determined by flow cytometry analysis. Representative data of two independent experiments were shown. (**c**) The effects of VentX on the expression of STAT1 and STAT3 were determined by qRT-PCR. Data shown are mean ± S.D. of three independent experiments, and paired Student's *t* test was performed. * $p < 0.05$.

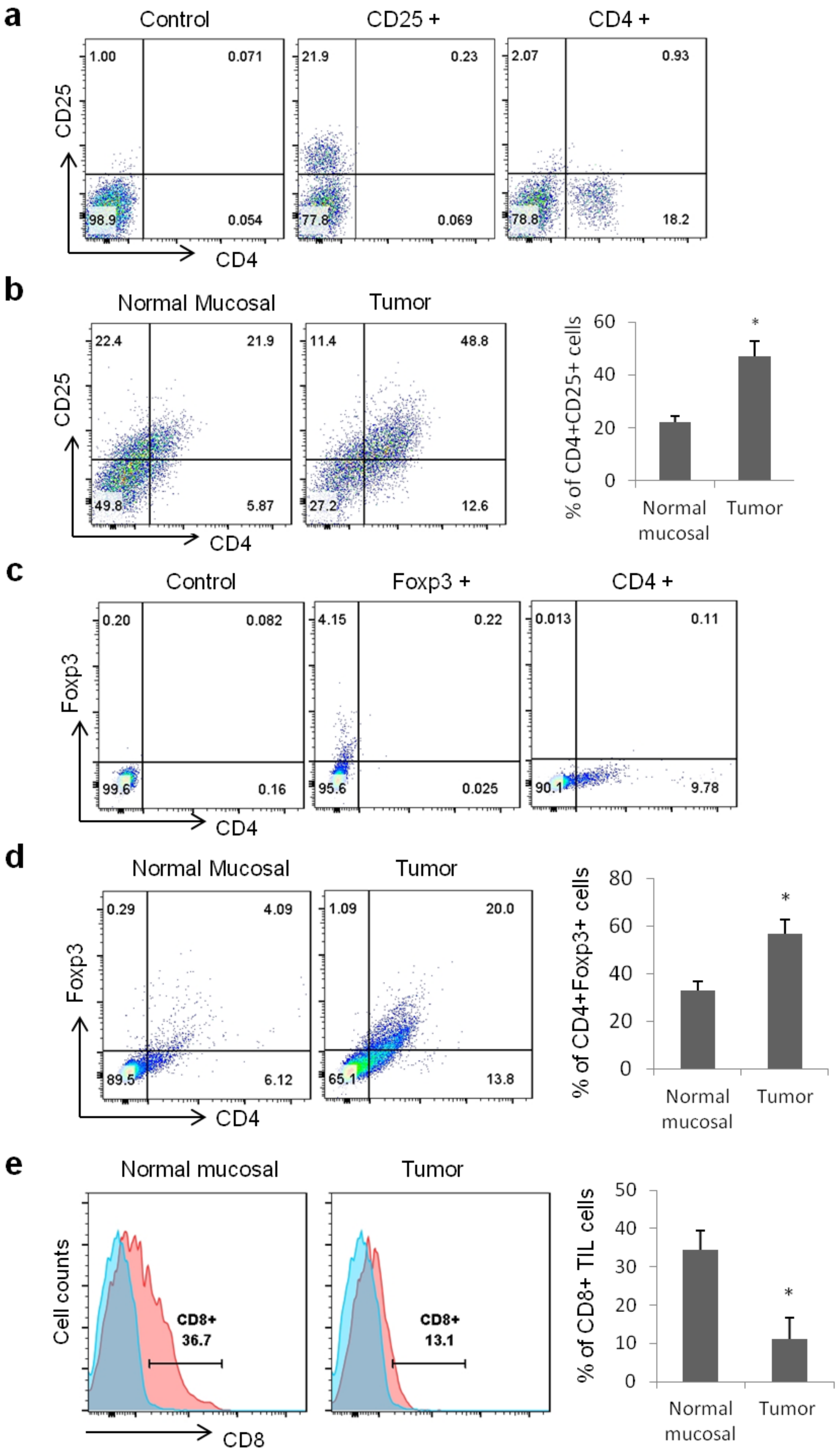
a
Control
CD25 +
CD4 +
CD25
CD4
1.00
0.071
98.9
0.054
21.9
0.23
77.8
0.069
2.07
0.93
78.8
18.2
b
Normal Mucosal
Tumor
22.4
21.9
49.8
5.87
11.4
48.8
27.2
12.6
% of CD4+CD25+ cells
Normal mucosal
c
Control
Foxp3 +
CD4 +
Foxp3
0.20
0.082
99.6
0.16
4.15
0.22
95.6
0.025
0.013
0.11
90.1
9.78
d
0.29
4.09
89.5
6.12
1.09
20.0
65.1
13.8
% of CD4+Foxp3+ cells
e
Normal mucosal
Cell counts
CD8
CD8+
36.7
13.1
% of CD8+ TIL cells

Supplementary Figure 6. Skewed T lymphocyte sub-populations in tumors vs normal tissues.

(**a**) Gating strategy of CD25 Treg cells. CD4 and CD25 single positive populations of normal tissues were demonstrated and used as standards for the gating. The gating strategy was applied for identifying CD4+CD25+ Treg cells in tumors vs normal tissues (**b**). (**c**) Gating strategy of Foxp3 positive Treg cells. CD4 and Foxp3 single positive populations of normal tissues were demonstrated and used as standards for the gating. (**d**) CD4+Foxp3+ Treg cells were determined by flow cytometry analysis in tumors vs normal tissues. (**e**) CD8 positive lymphocytes in tumors vs normal tissues were determined by flow cytometry analysis. Data shown are mean ± SD from 3 independent experiments, and paired Student's *t* test was performed. *$p < 0.05$.

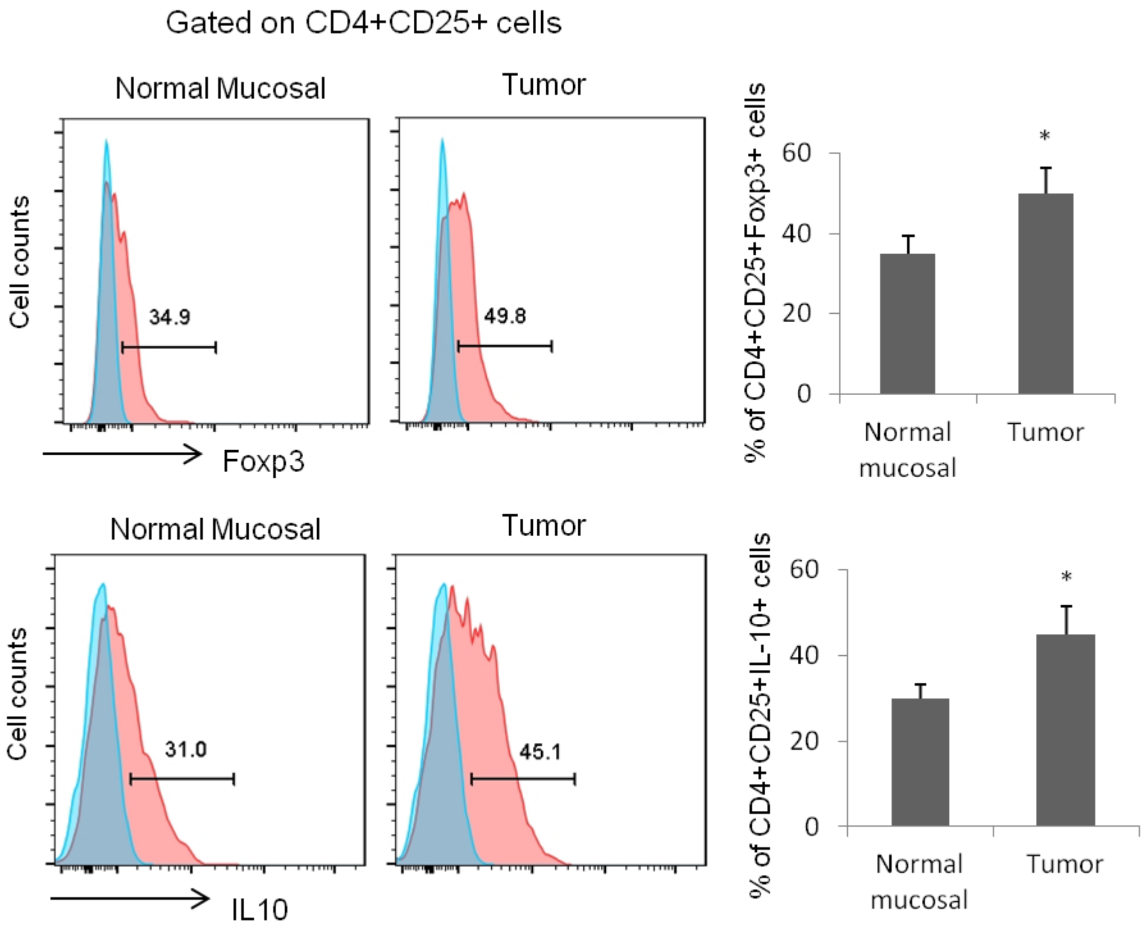

Supplementary Figure 7. Expression of FoxP3, IL-10 in CD4+CD25+ cells in normal and tumor tissues.

FACS analysis of Foxp3 and IL-10 in the CD4+CD25+ Treg cells isolated from normal mucosa and tumor tissues were carried out as described in material and methods. Sequential gating was applied on the CD4+CD25+ Treg cells for FoxP3 or IL10 makers as indicated. Data shown are mean ± SD from 3 independent experiments, and paired Student's *t* test was performed. *$p < 0.05$.

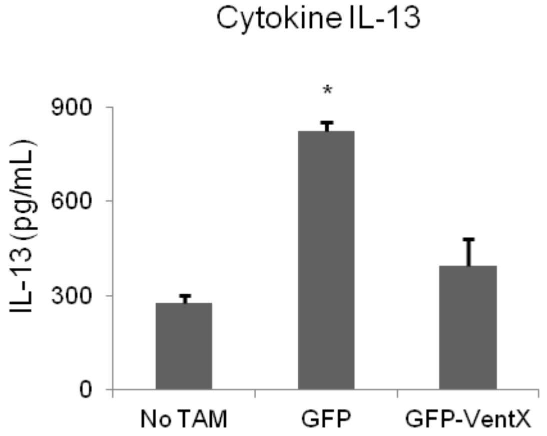

Supplementary Figure 8. VentX-regulated-TAMs affect cytokine secretion of T cells. CD4+ T cells were isolated from normal tissues and then incubated with GFP or GFP-VentX transfected TAMs for 5 days. The effects of the incubation on the secretion of T cell specific cytokine IL-13 were determined by ELISA assay. Data shown are mean ± SD from 3 independent experiments, paired Student's *t*-test was performed. *$p < 0.05$.

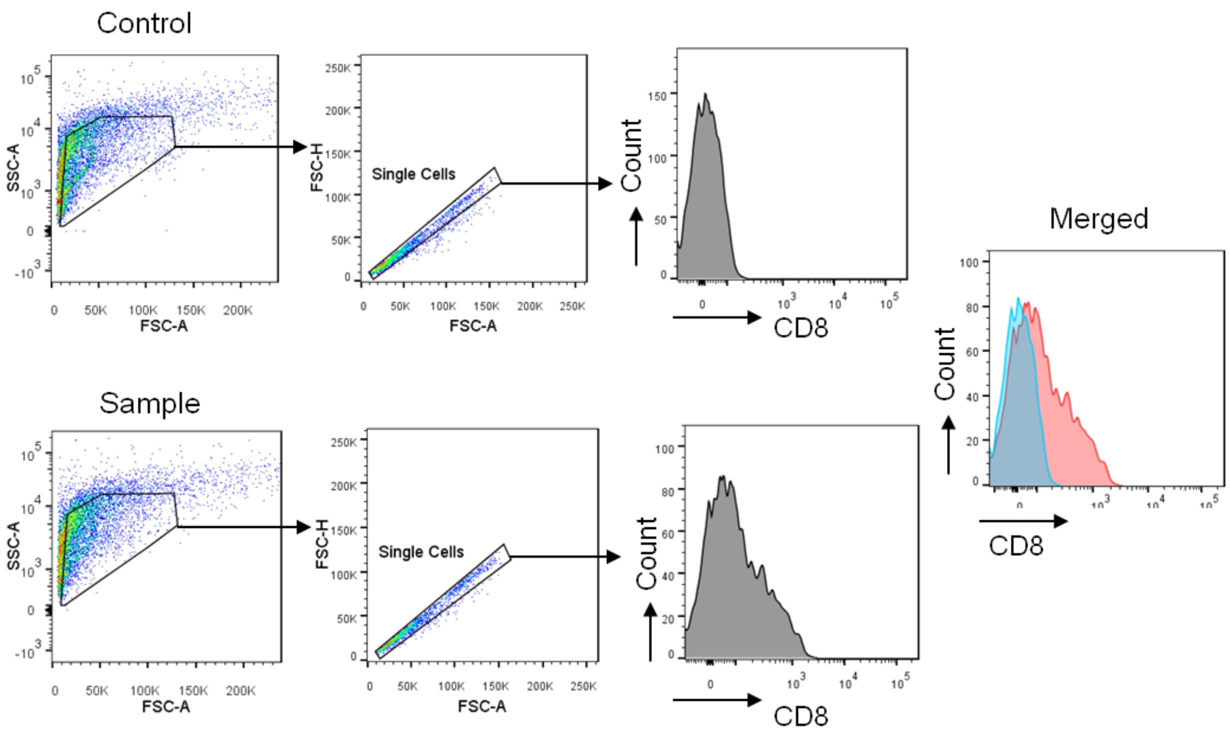

Supplementary Figure 9. Gating strategy for flow cytometry analysis of TIL CD8. Isolated LPMCs were surface stained with APC-conjugated anti-CD8 antibody and analysis with a flow cytometer. The lymphocyte populations were selected based on forward (FSC) and side scatter (SSC). Isotype antibody was used a negative control.

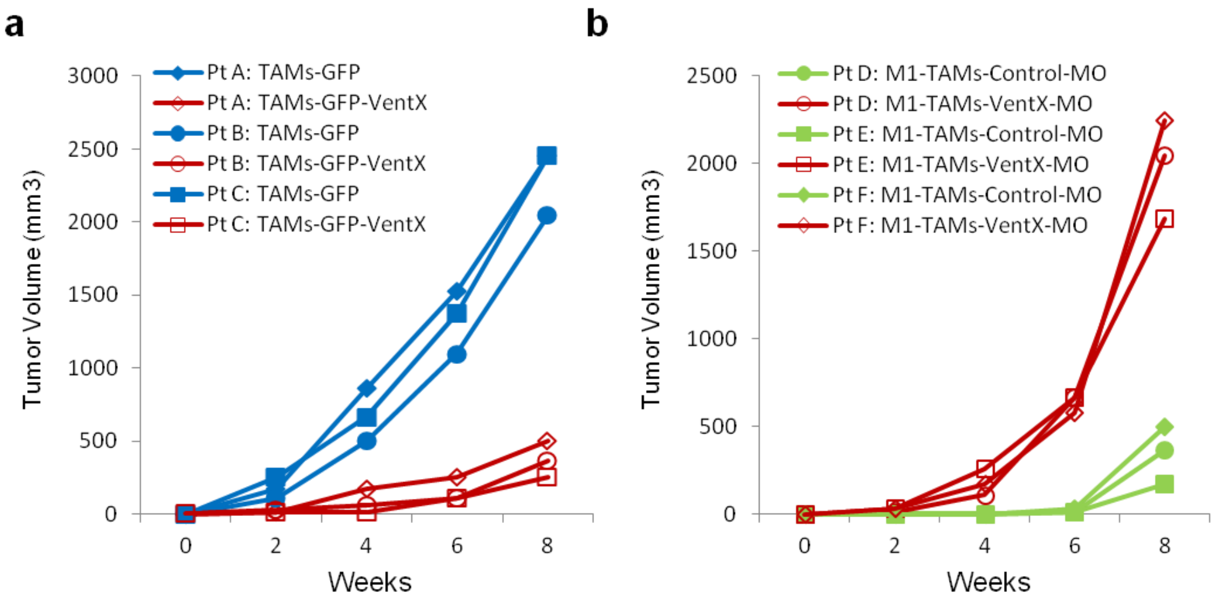

Supplementary Figure 10. Individual tumor growth curve in NSG-PDX mice.
(**a**) Individual growth curve of tumors in NSG-PDX mice treated with TAMs transfected with GFP-VentX or control GFP. (**b**) Individual growth curve of tumors in NSG-PDX mice treated with M1-TAMs transfected with VentX-MO or control MO.

a

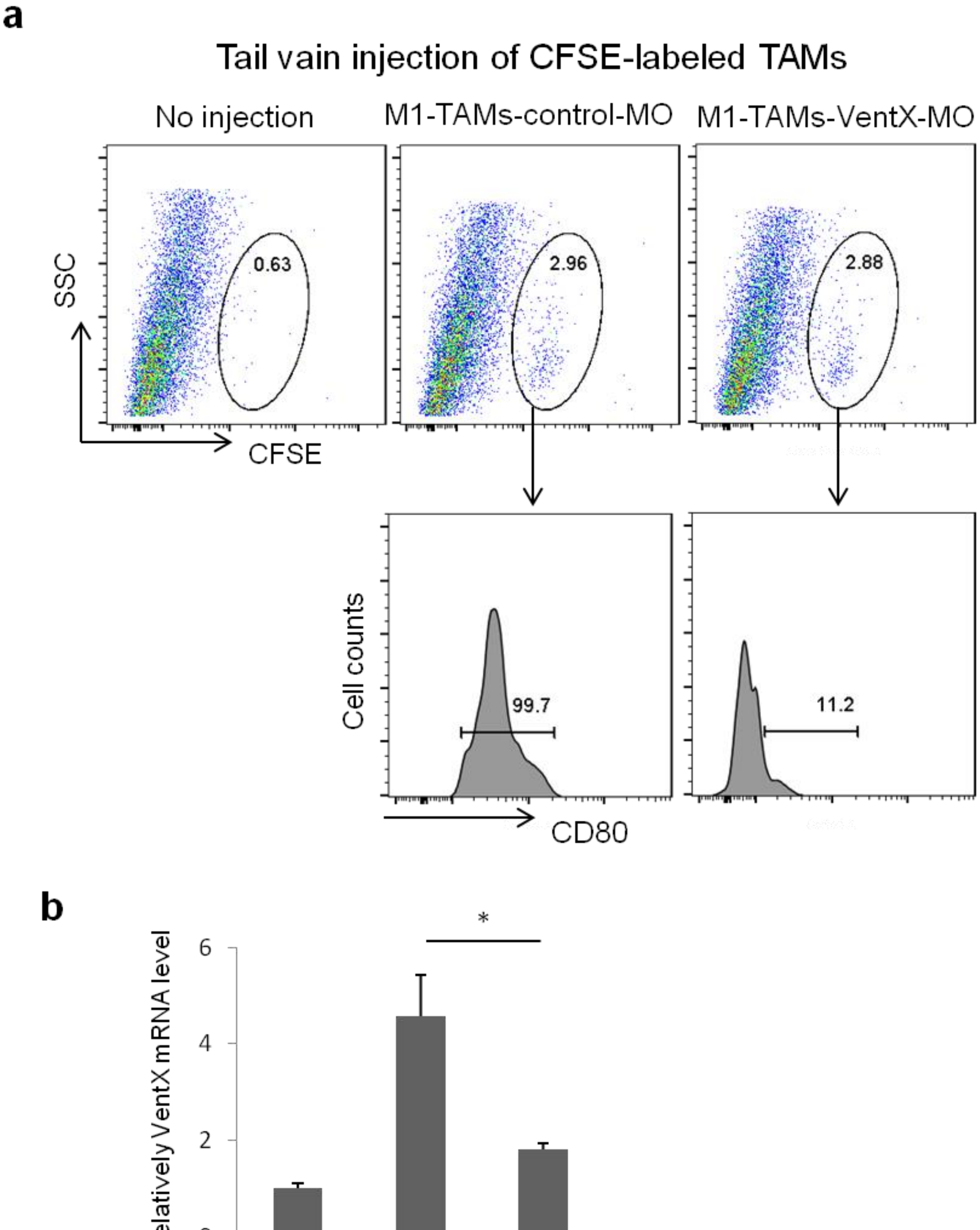

Tail vain injection of CFSE-labeled TAMs
No injection
M1-TAMs-control-MO
M1-TAMs-VentX-MO
SSC
CFSE
0.63
2.96
2.88
Cell counts
CD80
99.7
11.2
b
Relatively VentX mRNA level
6
4
2
0
*
TAMs
M1-TAMs Control-MO
M1-TAMs VentX-MO

Supplementary Figure 11. VentX modulate TAM plasticity *in vivo*.

M1 differentiated TAMs were transfected with VentX-MO or control MO and then labeled with CSFE as described in material and methods. The CSFE-labeled M1-TAMs were then tail-vein injected into NSG-PDX mice of human colon cancer. 72 hours post-injection, the tumors were removed from the mice and the TAMs were isolated using a ficoll gradient. (**a**) The CSFE positive cells were gated and the cell surface expression of M1 marker CD80 were determined by flow cytometry analysis. (**b**) VentX expression in the TAMs were determined by qRT-PCR. Data shown are mean ± SD of 3 independent experiments, and paired Student's *t* test was performed. *$p < 0.05$.

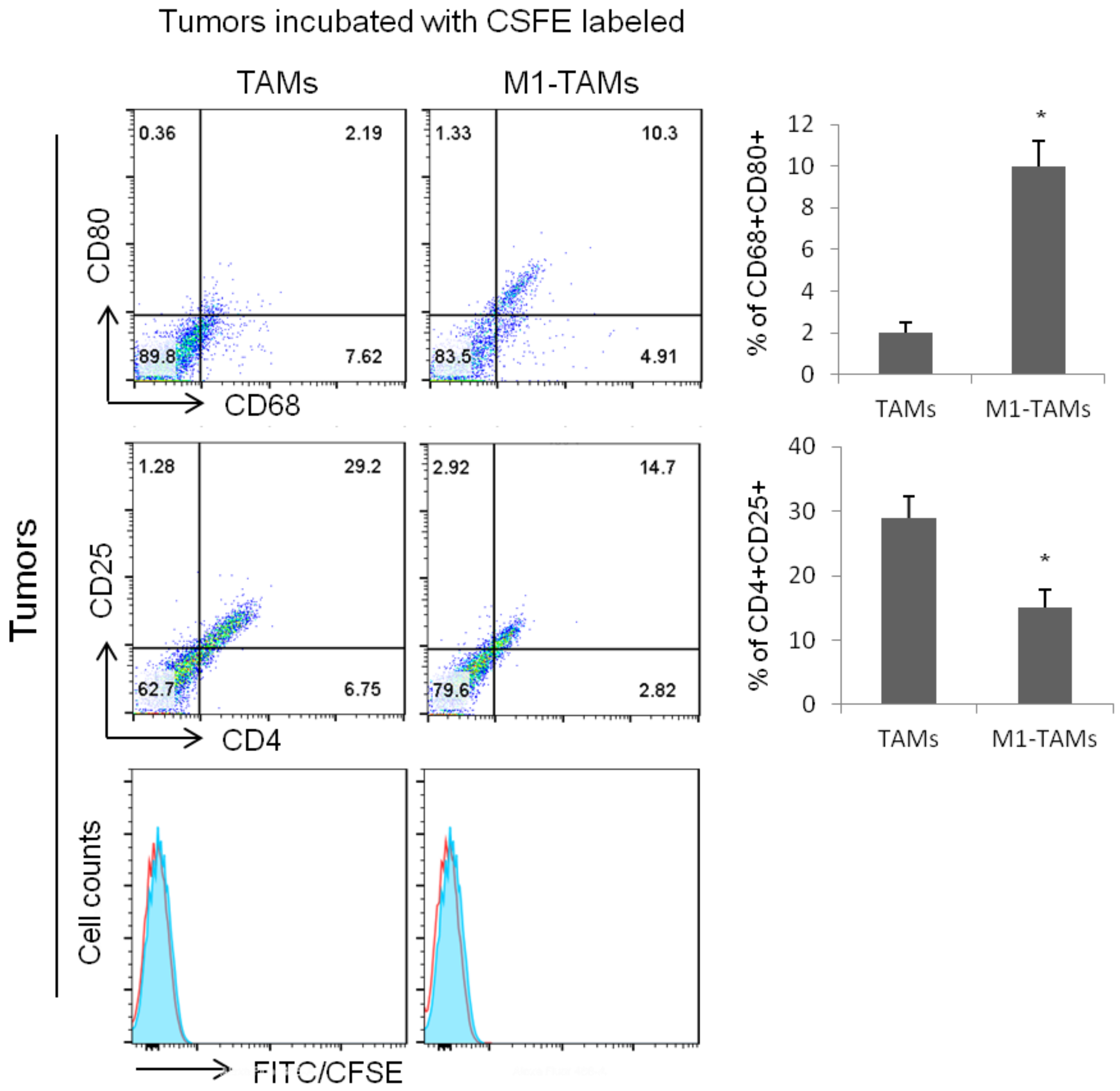

Supplementary Figure 12. Effects of exogenous TAM on tumor endogenous TAM and T cell differentiation.

En block tumor tissues were incubated with autologous CSFE labeled TAMs or M1-polarized-TAMs (M1-TAMs) for 5 days. Following the incubation, the tumors were isolated and washed extensively with RPMI and dissociated into single cell suspension as described in material and methods. The percentage of tumor endogenous M1-TAMs and Treg were determined by FACS analysis. Note, few CFSE-labeled TAMs accumulated inside the tumors during the incubation as indicated by the histogram of the lower panel. Data shown are mean ± SD from 3 independent experiments, and paired Student's *t* test was performed. $*p < 0.05$.

Supplementary Table 1

Primer sequences used in this study

Gene name	Forward	Reverse
VentX-C*	AAGGCAATTAGGCGCTGCTT	ACAGAACAACTGAGTCCTCCA
VentX-R*	CCGTCAGCATCAAGGAGG	CTGGACCTCTGAGAGCTGC
IL1β	AAGCTGATGGCCCTAAACAG	AGGTGCATCGTGCACATAAG
IL6	GAACTCCTTCTCCACAAGCGCCTT	CAAAAGACCAGTGATGATTTTCACCAGG
IL8	ATGACTTCCAAGCTGGCCGT	CCTCTTCAAAAACTTCTCCACA
IL12	GCAGAGGCTCTTCTGACCCCCA	AGCTGACCTCCACCTGCCGA
TNFα	CGC CAC CAC GCT CTT CTG	GCC ATT GGC CAG GAG GGC
CCL18	AGCTCTGCTGCCTCGTCTAT	CCCACTTCTTATTGGGGTCA
HIF-1	GCTGATTTGTGAACCCATTC	AAATTGAGCGGCCTAAAAGT
VEGFA	GGCAAAGTGACTGACCTGCT	CTGTCTGTCTGTCCGTCAGC
MMP-7	TGAGCTACAGTGGGAACAGG	TCATCGAAGTGAGCATCTCC
MMP-9	TTGACAGCGACAAGAAGTGG	GCCATTCACGTCGTCCTTAT
Arginase1	ACAGTTTGGCAATTGGAAGCA	CACCCAGATGACTCCAAGATCAG
iNOS	GCAGAATGTGACCATCATGG	ACAACCTTGGTGTTGAAGGC
STAT1	CCATCCTTTGGTACAACATGC	TGCACATGGTGGAGTCAGG
STAT3	GCTTTTGTCAGCGATGGAGT	ATTTGTTGACGGGTCTGAAGTT
	C*: conventional PCR; R*: real-time PCR	
